# 3D Bioprinting at the Frontier of Regenerative Medicine, Pharmaceutical, and Food Industries

**DOI:** 10.3389/fmedt.2020.607648

**Published:** 2021-01-28

**Authors:** Qasem Ramadan, Mohammed Zourob

**Affiliations:** College of Science and General Studies, Alfaisal University, Riyadh, Saudi Arabia

**Keywords:** 3D bioprinting, tissue engineering, regenerative medicine, drug discovery, *in vitro*

## Abstract

3D printing technology has emerged as a key driver behind an ongoing paradigm shift in the production process of various industrial domains. The integration of 3D printing into tissue engineering, by utilizing life cells which are encapsulated in specific natural or synthetic biomaterials (e.g., hydrogels) as bioinks, is paving the way toward devising many innovating solutions for key biomedical and healthcare challenges and heralds' new frontiers in medicine, pharmaceutical, and food industries. Here, we present a synthesis of the available 3D bioprinting technology from what is found and what has been achieved in various applications and discussed the capabilities and limitations encountered in this technology.

## Introduction

Additive manufacturing (AM), the process of joining materials to make objects from computer-aided design (CAD) model data, such as 3D printing, shows a high potential to radically disrupt the global consumer market and trigger a manufacturing revolution in a broad spectrum of applications in many industry sectors. 3D printing is mostly well-known for custom-fabricating of industrial prototypes and parts using standard fabrication materials such as plastics and metals. This technology has recently infiltrated into many industries such as aviation, automobile, dental, electronics, and fashion. The successful implementation of AM in the healthcare industry has resulted in the development of surgical equipment, prosthetics, medical devices, and implants. More recently, 3D bioprinting technology has emerged from the existing 3D printing, by utilizing life cells and gels as printing materials (bioinks) to create *ex vivo* and *in vitro* tissue models, which heralds' new frontiers in medicine.

3D bioprinting is the process of integrating living cells with biomaterials that allows controlled layer-by-layer deposition of cells/bioink, which is characterized by hierarchical structural properties, with maintained cellular viability in 3D space to create complex, multifaceted tissues. 3D bioprinting benefited from several technologies such as tissue engineering, synthetic biology, micro/nanofabrication, and bioprocessing biomaterial production (1).

**Figure 1:**
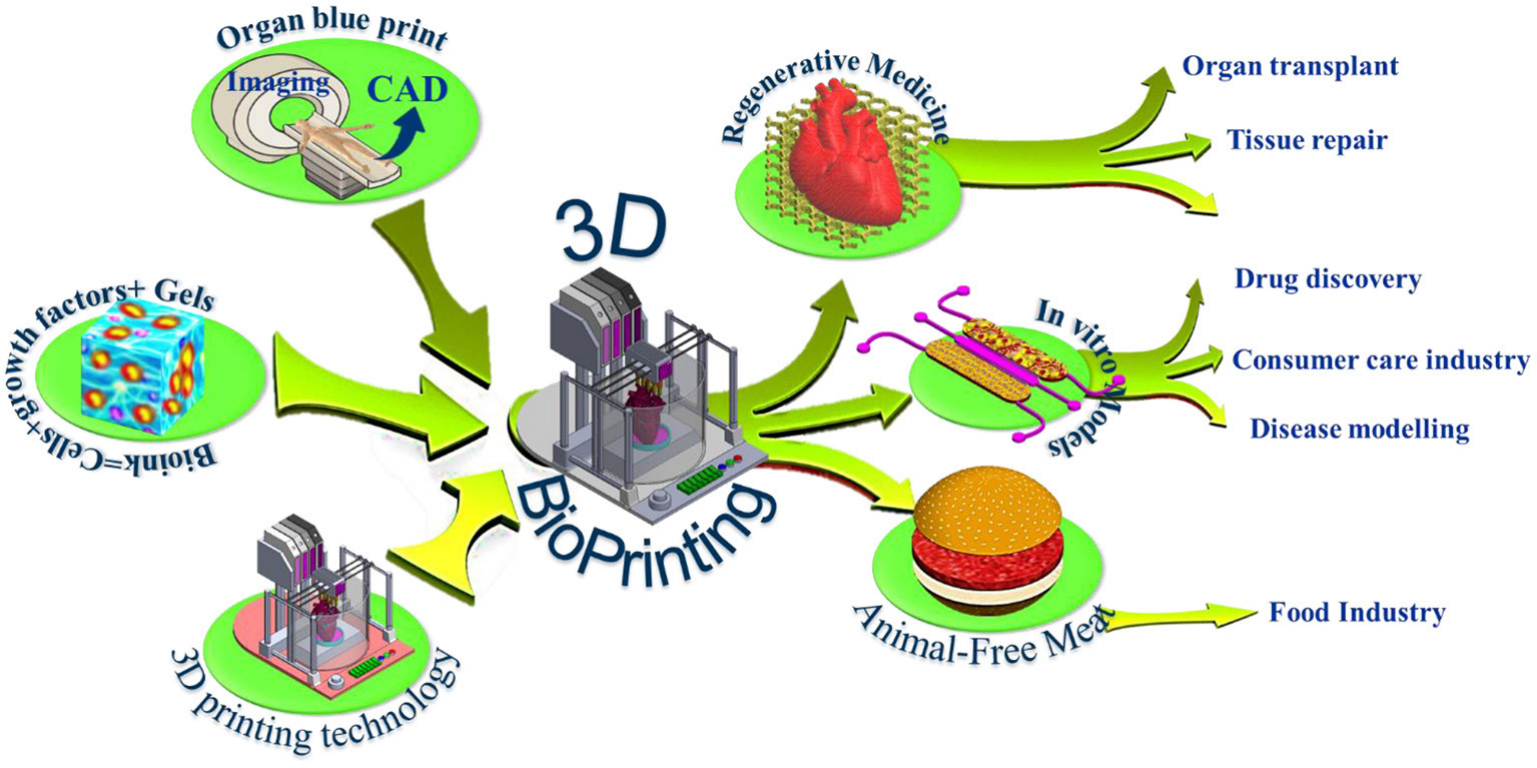
3D bioprinting integrates the conventional 3D printing, imaging, and cell-gel to fabricate functional tissue for regenerative medicine, pharmaceutical preclinical drug screening, and animal-free meat.

*In vitro*, cells cannot arrange themselves in 3D structures like that in real tissue *in vivo*. Various techniques were utilized and developed, aiming to mimic the living tissue structure and function, such as scaffold fabrication, tissue culture, bioreactors, ECM self-assembly among others. However, current tissue-engineering strategies lack the capability of fabrication of fully functional tissues and recapitulate their heterocellular structure (2, 3). 3D printing shares the three basic components with the conventional Gutenberg paper printing: the 3D model file to be printed (blueprint) is analogous to the text file, the bioink (which comprises cells and other bioactive materials) is analogous to the ink, and the 3d printer is analogous to the printer that deposit the ink on a substrate or print platform (Figure 1). The progress in bioprinting technology is going separately through these techniques, and the real challenge is to integrate these technologies in an industrial scalable technology (1). However, bioprinting has a great potential to surpass the traditional tissue engineering techniques and can offer solutions to many existed technological hurdles such as:

- 3d bioprinting allows a high level of control and precise positioning of several cell types, thanks to the precise control position of the dispenser nozzle in the X–Y–Z space (1, 4), hence enabling accurate recapitulation of tissue/organ microstructure with high architectural complexity.
- 3d bioprinting is amenable to automation and scalable technology that would enable the mass production of tissue/organ from standard building blocks (4).
- 3d bioprinting benefits from the well-established printing technology. Therefore, the engineering of the modular 3d bioprinter is advancing with high-speed pace. For example, the 3d bioprinter could be evolved as an integrated surgical tool for *in situ* printing (5).

The last decade witnessed significant progress in the bioprinting arsenal with many ground-breaking innovations that makes 3D bioprinting one of the most exciting and promising technologies that could impact many medical applications. Using bioprinting technology, scientists may print living *de novo* organs like heart, livers, kidneys, lungs, and skin, which would, therefore, reduce the organ transplant shortage. At the same time, when cells are taken from the patient himself, this would ensure eliminating the immune system attack and organ rejection. Another exciting industrial application of 3d bioprinting is in the pharmaceutical industry. *In vivo*-like *in vitro* models can be printed using human cells, and a living organ or a network of organs can be created and utilized for preclinical drug screening as an animal alternative. Another exciting application is using the 3d printing technology and advanced food formulations to produce animal-free meat that mimics the appearance, texture, and taste of animal-based meat (Figure 1). These three applications hold great potential in creating new markets and form the major driving forces that accelerate the research and development in academia and industry. The industry sector of this domain is expanding rapidly with many businesses having been established that centered around this emerging technology such as Organovo Holdings, Inc. (US), CELLINK (Sweden), Allevi Inc. (US), Aspect Biosystems Ltd. (Canada), EnvisionTEC GmbH (Germany), Cyfuse Biomedical K.K. (Japan), Poietis (France), TeVido BioDevices (US), Nano3D Biosciences, Inc. (US), ROKIT Healthcare (South Korea), Digilab, Inc. (US), regenHU (Switzerland), GeSiM (Germany), Advanced Solutions Life Sciences (US), and Regenovo Biotechnology Co., Ltd. (China), Hewlett-Packard (Palo Alto, CA, USA), Novogen MMX Bio-printer (Organovo, Inc., San Diego, CA, USA), 3D Bioplotter (EnvisionTEC, Gladbeck, Germany), Oxford Performance Materials (South Windsor, CT, USA), and Commercial Blood Vessel Bioprinter (Revotek, Sichuan, China), among others. The 3D bioprinting market is projected to reach USD 1,647.4 million by 2024, driven by the technological advancements in 3D bioprinters and biomaterials and its application in the pharmaceutical and cosmetology industries (6). The Food and Drug Administration (FDA) issued a guidance that provides the Agency's initial thinking on technical considerations specific to devices used for 3D printing techniques and products (7).

Many technical challenges are still ahead and need to be solved to enable smoother penetration of this technology into the market. Several excellent reviews were recently published (8–13) which surveyed the landscape of 3D bioprinting. In this paper, we briefly presented the current bioprinting techniques and other essential elements pertaining to the application of 3D bioprinting for generating 3D tissues/organ. We also discuss the major challenges and exciting opportunities of 3D bioprinting technologies toward creating realistic tissue/organs in various market sectors particularly on the potential of creating *in vitro* models as tools of drug discovery in the pharmaceutical industry.

## 3D Bioprinting Technology

**Figure 2:**
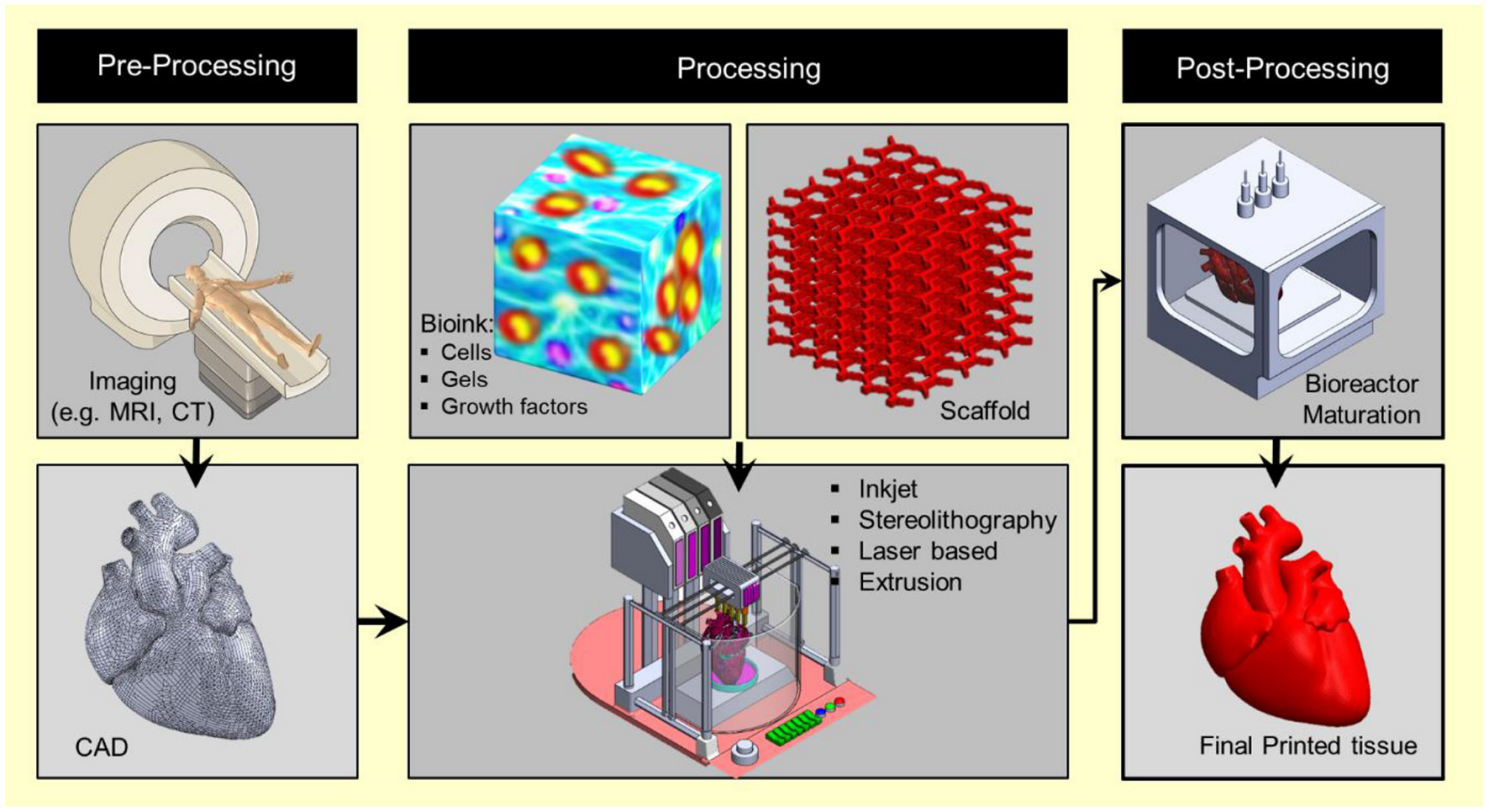
Overview schematic of the bioprinting processes.

Bioprinting starts with obtaining the anatomical structure of the target tissue by a proper imaging technique such as computerized tomography (CT) and magnetic resonance imaging (MRI). A specialized software is then used to translate the image to a CAD drawing of cross-sectional layers such that the printing device will be able to add them in a layer-by-layer process. Next, the bioprinting device constructs the tissue using a specific printing method such as inkjet 3D bioprinting, micro-extrusion 3D bioprinting, laser-assisted 3D bioprinting, and stereo-lithography by employing a combination of printing materials such as scaffold, bioink, and other additive factors (Figure 2). The accuracy, stability, and tissue viability vary through these processes. Finally, the constructed tissue is post-processed in a bioreactor to recreate the required *in vivo* environment to maintain tissue viability during the maturation period.

### 3D Bioprinting Techniques

3D bioprinting technology was evolved from the traditional 2D printing on paper and later 3d printing of non-biological materials. Therefore, it is not surprising that the engineering aspect is more advanced than bioink material technology. However, since it was initially developed for non-biological material printing, each printing technology is still suffering from several limitations related to material compatibility when replacing other building materials with bioink. Several reviews have been recently published which provide comprehensive technical information on these techniques (8–14). Therefore, here we only briefly discuss these techniques, which are also summarized in Table 1.

- **Microextrusion 3D bioprinting**: is a pressure-assisted technique commonly used in non-biological material printing. In the bioprinting process, the selected bioinks, which are stored in a glass or plastic cartridge, are normally dispensed through a nozzle by applying pressure using either a pneumatic or mechanical (piston or screw-driven) method and controlled by a computerized robotic arm. The bioink is ejected through the nozzle in the form of a thin filament and deposited on the substrate based on a CAD design that determines the position and path of nozzle movement to form the tissue in the desired 3d shape. This technique originated from conventional 3D printing and has been found to be the most suitable method for the creation of large-scale constructs, due to its structural integrity, hence becoming more amenable to scale-up for organ fabrication (12). In addition, a large variety of bioinks can be used, including scaffold-based and scaffold-free bioinks, with high printing speed. However, this technique has a low resolution (~100 μm) (14, 29). The relatively high extrusion pressure through the nozzle imposes high shear stress on the bioink components and may lead to loss of cellular viability and distortion of the tissue structure (16, 30).
- **Inkjet 3D bioprinting**: is a non-contact technique that uses thermal, piezoelectric, or electromagnetic forces to expel bioink droplets onto a substrate replicating the CAD-based model. This technique originated from the conventional and well-established 2D paper-based printing, which makes it a low-cost approach (19, 20). Other key advantages of inkjet bioprinting include high-speed printing with multiple nozzles (21, 22), which may enable the fabrication of heterogeneous tissue structures (22) and the relatively high cell viability (24). On the other hand, inkjet bioprinting requires bioink with low viscosity (<0.1 Pa S-1) (31), making the deposition of highly viscous hydrogels and ECM more difficult.
- **Laser-assisted 3D bioprinting (LAB):** is also a non-contact printing technique. The laser-assisted bioprinter uses a ribbon, which is coated with an absorbing layer such as gold. When a laser pulse is directed and passed through the transparent ribbon, the generated heat induces a hydrogel droplet and is eventually transferred to the receiving substrate. This process is repeated several times, through laser pulses, to form a jet, consequently creating the final construct in a layer-by-layer fashion. Using this technique, cells can be deposited with a density of up to 10^8^ cells/ml with a single-cell resolution at high speed, enabling high-throughput cell and biomaterial patterning (24–26). In addition, laser-assisted printing offers real-time monitoring of cells, therefore enabling cell selection and transfer (5). On the other hand, the excessive heat generated due to the laser energy may damage cells and affect the cell viability in the printed tissue (32).
- **Stereolithography-based bioprinting (SLB):** utilizes photopolymerizable liquid polymer where a UV light or laser is directed in a predesigned pattern over the polymer, which leads to cross-linking and hardening of the polymers. In every polymerization cycle, a thin layer of the structure is created, and this polymerization cycle is repeated to build the 3D structure in a layer-by-layer fashion. The main advantage of this technology lies in its high resolution and the absence of harsh shear stress compared to other techniques. However, cells are exposed to intense UV radiation for cross-linking, which can cause cell damage.

**Table 1:** Major 3D bioprinting techniques.

**Bioprinting technique**	**Description**	**Advantages**	**Disadvantages**	**References**
Microextrusion 3D bioprinting 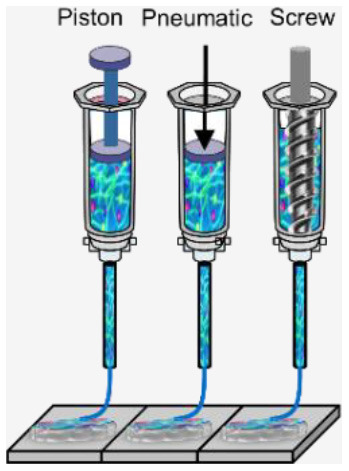	Continuous dispensing of the printing materials (bioink) through a nozzle that is driven by a pneumatic or mechanical (piston or screw-driven) method and controlled by a computerized robotic arm	• The ability to print high-viscosity bioinks by adjusting the driving pressure; • The ability to print tissues with very high cell densities and scaffold-free bioink; • Provides good structural integrity due to the continuous deposition of filaments • Amenable to scale-up tissue and organ fabrication process	• The pressure-driven dispensing results in high shear stress on the cells, which dramatically affects the cell viability; • Limited resolution; inability to construct a microcapillary network	(14–18)
Inkjet 3D printing 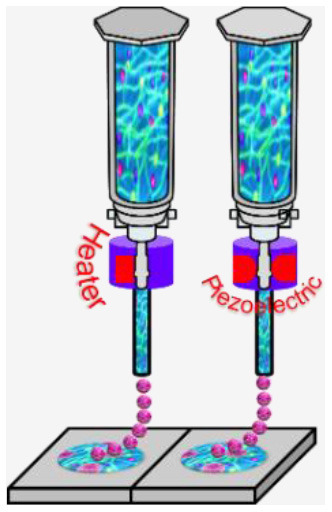	Droplets of cell-containing bioink (each contains 10,000–30,000 cells) is formed by either heating or piezoelectric through a non-contact nozzle	• Non-contact based, which reduces the chance of contamination; • The ability to integrate multi-printing heads for heterogeneous tissue structures; • Enables fabrication of a vasculature-like structure; • High-speed printing and amenable to high-throughput printing	• Non-uniform droplet size; • Requires bioink with low viscosity (<0.1 Pa s^−1^). This makes the deposition of highly viscous hydrogel ECM components more difficult	(19–23)
Laser-assisted 3D bioprinting 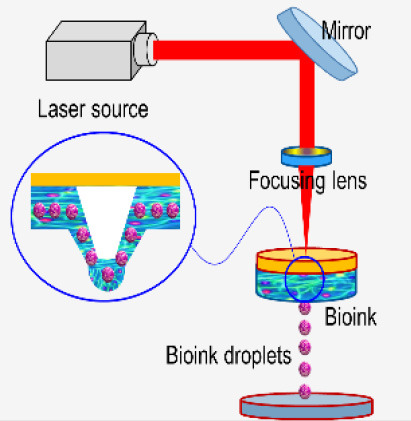	A focused laser pulse creates a bubble and shock waves that force cells to transfer toward the collector substrate. The step is repeatedly performed to create predesigned 3D structures	• High precision and resolution for the printed structures which make it possible for bioprinting of micro-patterned peptides, DNA, and cells with single-cell resolution; • The ability to print tissues with very high cell densities; • No viscosity limitations	The heat generated from laser energy may affect the cell viability	(24–26)
Stereolithography-based bioprinting 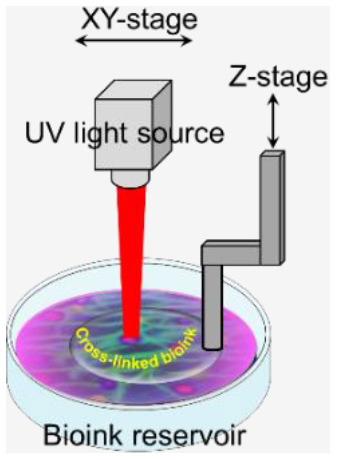	UV light or laser is directed in a pattern over a photopolymerizable polymer or bioink that leads to cross-linking of the polymers into a hardened layer to eventually form 3D object/tissue	High resolution; no clogging during the printing process	• Needs an intense radiation for the cross-linking; • Slow process	(27, 28)

### Bioinks

The development of printing biomaterials (i.e., bioink) is a cornerstone of 3D bioprinting technology and the most challenging task that is still delaying this technology's progress. The ideal properties for bioink must meet both the physical and biological material requirements to enable *in vivo*-like cellular behavior, such as proliferation, differentiation, migration, and maturation. The physical properties are viscosity, structural strength, printing capacity, degradation, and functionality. Biological properties include cytocompatibility, biocompatibility, and bioactivity (16). Bioink viscosity is a crucial parameter of the bioprinting process that always needs optimization to adjust the bioink flow and cell encapsulation efficiency, and tissue structure stability (16). Diverse bioink compositions existed to meet the requirements of specific printing technology. Hydrogels are promising candidates for developing of bioinks thanks to their biocompatibility, low cytotoxicity, hydrophilicity, and ability to form networks of polymers allowing them to acquire ECM with a similar structure.

Tissues and organs are self-organizing systems. During the embryonic maturation process, cells undergo biological self-assembly and self-organization without external influence or guiding structures (33). However, *in vitro*, the cell microenvironment is dramatically simplified or does not exist; therefore, cells fused and slowly aggregate differently. In bioprinting, biocompatible scaffolds are usually used as structural support for cells to adhere, proliferate, differentiate, and eventually form the tissue. Studies showed that the arrangement of integrins within a scaffold highly influenced stem cell differentiation (34). Recapitulating the native environment of a specific tissue type promotes stem cell differentiation toward that lineage. The ability to create 3D heterogeneous tissue structures relies on the integration with compatible biomaterials to support the cellular components. Hydrogels are the most common biomaterials for live-cell printing (5, 35) owing to their biocompatibility and their ability to acquire a similar structure the ECM (5, 35–37).

**Figure 3:**
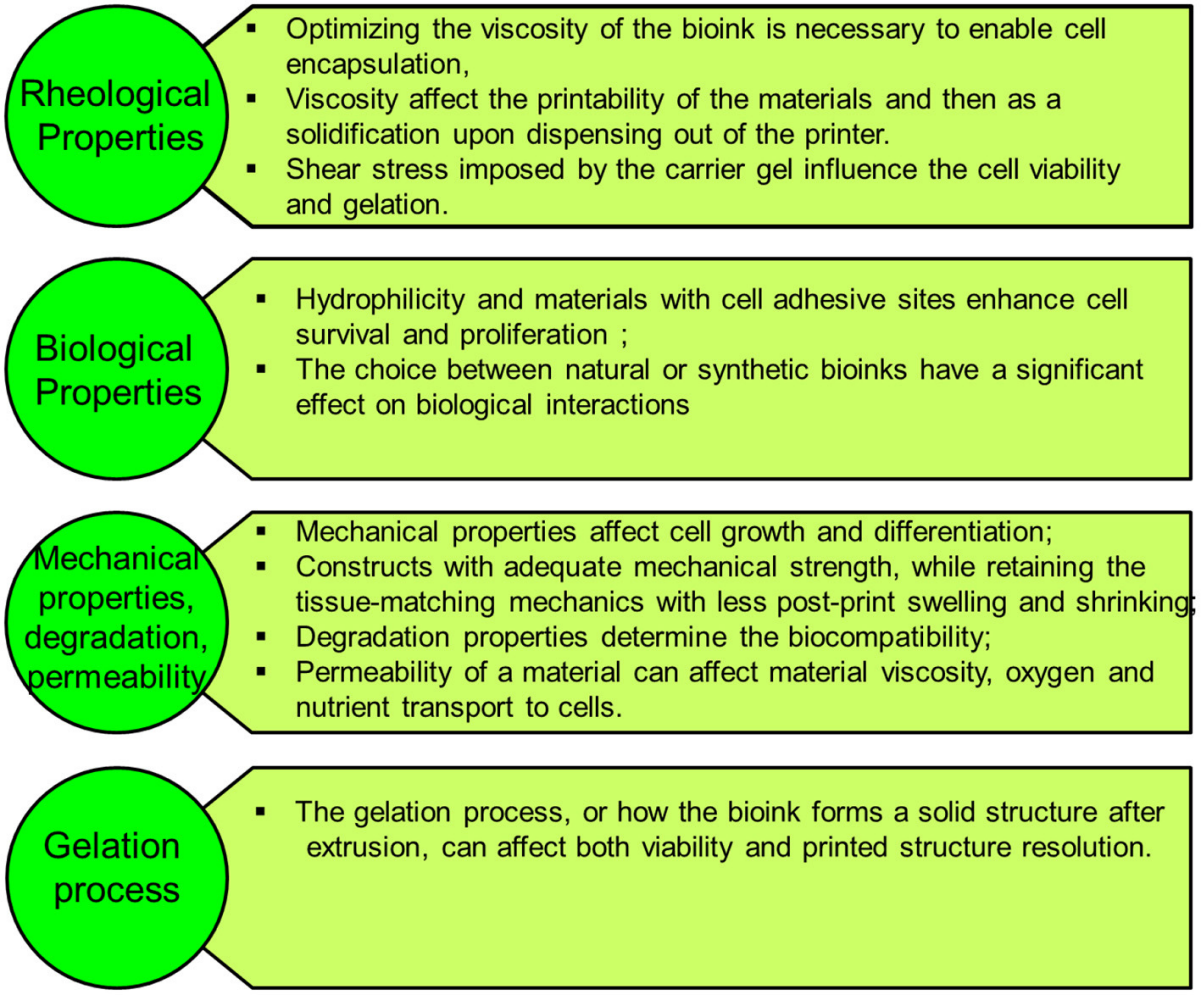
The bioink matrix properties play a vital role in the effectiveness of the bioink in the bioprinting process and for creating viable 3D tissues with complex geometries.

The ultimate aim of bioprinting-based tissue engineering is to mimic to a certain extent the embryonically developed tissue/organs. However, this novel approach is still not close enough to achieve the same degree of complexity of the *in vivo* counterparts produced by different specialized cells and dynamic extracellular matrix (ECM) composition (9). Bioink is a solution of a biomaterial or a mixture of several biomaterials (e.g., in a hydrogel form), which encapsulates the desired cell types during the printing process to create the tissue constructs. Bioinks are made of either natural or synthetic biomaterials, or a mixture of both (38). The biological, mechanical, and rheological properties of bioinks should be optimized to enable creating the tissue that closely mimics the structure and functions of the *in vivo* counterpart. Different applications and cell types may require different bioinks. In general, there are essential properties that need to be considered when choosing a bioink, such as the following (Figure 3):

- **Viscosity**: Depending on the bioprinting technique, the bioink matrix should fit in all the bioprinting phases, as fluid during cell encapsulation and as a solid after dispensing (39);
- **Gelation process and stabilization**: the process of how the bioink forms a solid structure after extrusion can affect the viability and printed structure resolution. This process should be fast, and the bioink should retain the tissue-matching mechanics after printing and be non-toxic to cells. Various gelation processes are used, which may be determined by bioink material properties and composition, such as ionic (36), thermal (40), stereocomplex (29), photocrosslinking (41), enzymatic (42), and click chemistry (43);
- **Biocompatibility**: hydrophilicity and materials with cell-adhesive sites enhance cell survival and proliferation (44). Also, the choice between natural or synthetic bioinks has a significant effect on biological interactions. Natural-based bioinks may withstand harsh fabrication conditions (e.g., high temperatures and organic solvents); however, it suffers from batch-to-batch variability. On the other hand, synthetic polymers overcome the batch-to-batch variability, which offer a high potential for large-scale production but do not offer the natural cell adhesive sites (44).
- **Mechanical properties**: Cells are sensitive to their external mechanical environment, such as matrix elasticity (45), and can modify their behavior. Therefore, controlling the mechanical parameters of bioinks can be exploited to control cell behavior, such as their morphology and rate of proliferation, within the printed tissue construct, which plays an important role in the generation of a functional tissue. Another important mechanical property is shear-thinning, which is a non-Newtonian behavior that implies decrease of the viscosity as the shear rate increases, which causes reorganization of the polymer chain (46). For example, Chen et al. (47) developed a shear-thinning hybrid bioink by combining rigid gellan gum, flexible sodium alginate, bioactive thixotropic magnesium phosphate-based gel, and thixotropic TMP-BG. The bioink mechanical, rheological, and bioactive properties were optimized for printability and cell viability.

Various natural and synthetic biomaterials have been utilized as bioinks. Recently, responsive, dynamic, and supramolecular materials are being exploited for bioink development (48). Morgan et al. (11) provide a comprehensive review on the current trends in bioinks including the mechanical properties and dynamic bioinks. Synthetic polymers have good potential to be modified to induce bioactivity (38, 49). However, they may generate toxic products and lose their mechanical properties during the degradation process (50). In addition, self-assembling peptides are promising biomaterials for building 3D scaffolds that share similar structural and mechanical properties of extracellular matrices. For example, Cofiño et al. (51) developed bioink with controlled viscosity by optimizing methylcellulose and RAD16-I-based biomaterial to build 3D predefined structures. The resultant constructs show high shape fidelity and stability. In general, standardized bioink formulations are urgently needed to allow utility in different bioprinting applications.

### Cell Aggregates as Building Blocks

Using a biodegradable solid scaffold is, generally, the dominated approach in tissue engineering to construct a living tissue structure, which provides several supporting functions, including (1) a substrate to cell growth and proliferation; (2) a rigid scaffold to provide the desired tissue/organ shape; and (3) a porous structure of a solid scaffold which allows good cell viability and enables vascularization (33). However, this classical approach faces some limitations and challenges, which include (1) vascularization of thick tissue constructs, (2) precise positioning of multiple cell types inside the 3D scaffolds, and (3) the effect of scaffold rigidity on cell differentiation (45). The ideal alternative to solid scaffold techniques is to understand how organs are formed during embryonic development, which would provide a powerful insight into tissue engineering (33, 52). Researchers are recently looking at spherical cell aggregates (cellular spheroid) as building blocks of tissue construction. This development-biology-inspired approach involves utilizing self-assembly of these living microstructures to build tissues of prescribed shapes (53). During the embryonic maturation process, cells from multiple sources undergo biological self-assembly and self-organization without any external influence (33). In bioprinting (*in vitro*), cell aggregates undergo tissue fusion, where cells organize into multicellular units to create the final tissue structure. Forgacs et al. (53), showed that the fusion of embryonic cushion tissue during heart morphogenesis proceeds similarly *in vitro* and *in vivo* and both qualitatively and quantitatively resembles the coalescence of liquid drops and showed that spherical cell aggregates mixed with an appropriate hydrogel behave as self-assembling “bio-ink” particles. The authors also demonstrated the print of cellular toroid, tubes, and “beating” sheets of cardiomyocytes. Cell aggregates can be homogeneous (single-cell type) or heterogeneous (several cell types) and can be prepared using different methods (33, 54) such as hanging drop plates (55), ultra-low attachment (ULA) plates coated with hydrophilic hydrogel (56), and surface coatings that mimic the basement membrane and extracellular matrix (57), among others. Various organoid models are available^1^, which demonstrates the scalability of this technology and makes attractive to be adopted as large-scale industrial bioprinting and tissue/organ engineering industry.

## Applications of 3D Bioprinting

### 3D Bioprinting for Organ Transplanting

**Figure 4:**
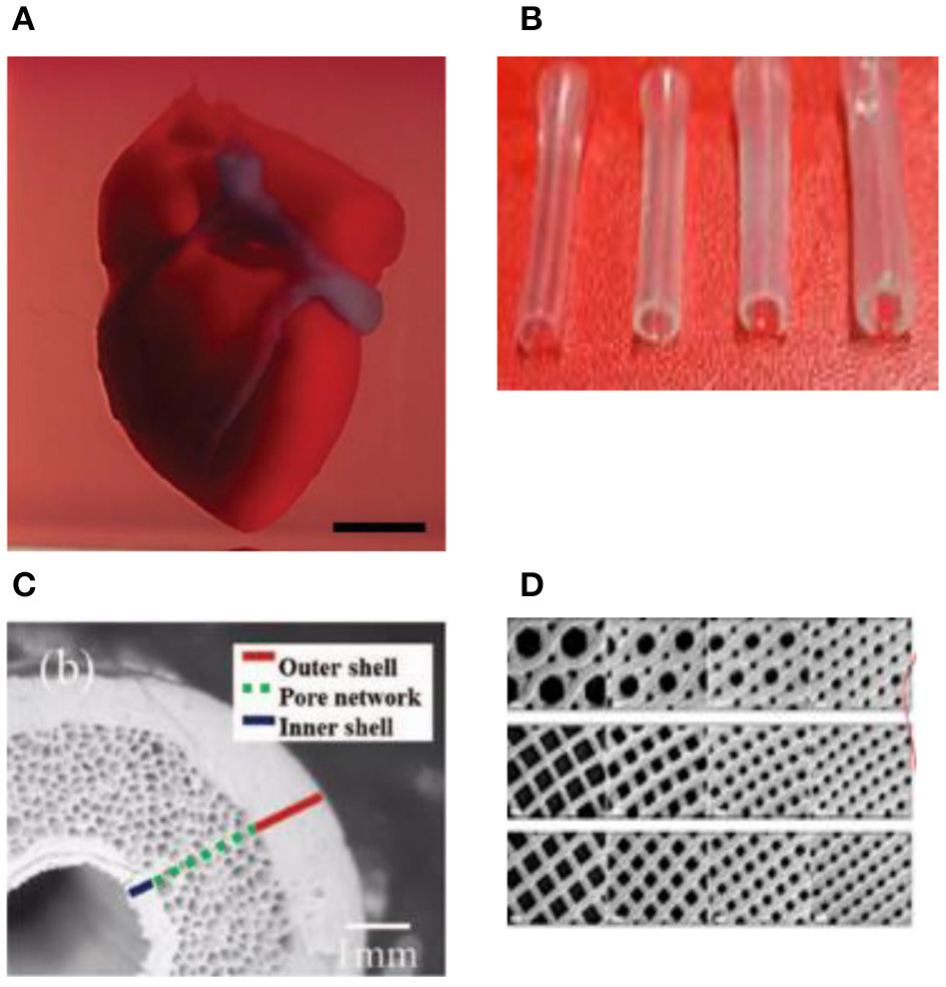
**(A)** A proof of concept of a cellularized human heart with a natural architecture is printed using microextrusion 3D bioprinting. Reproduced from Zhuang et al. (62). Open access (BB-CY). **(B)** Vessel-like structures printed utilizing alginate–gelatin solution. Reproduced from Liu et al. Open access (BB-CY) (65). **(C)** A porous hydroxyapatite scaffold with unidirectional microchannels at the exterior part of the scaffold to facilitate biomineralization and a central canal that houses the bone marrow. Reproduced from Jang et al. (66). Open access (BB-CY). **(D)** An anisotropic glass-ceramic scaffold with a mechanical strength comparable to cortical bone to repair large bone defects. Reproduced from Roohani-Esfahani et al. (67). Open access (BB-CY).

Leveraging on the tremendous success of printing industrial prototypes to prosthetics and surgical instruments, 3D bioprinting technology shows excellent progress in creating thick living cellular structures as an intermediate stage toward organ-level complexity. Despite the limitations with the associated biology and engineering, bioprinting holds great promise in whole-organ printing with an excellent hierarchical arrangement of cells and building tissue blocks in a 3D microenvironment. To print living tissues, cells are taken from either patient or adult stem cells and cultivated into a bioink. These ingredients are held together through a dissolvable gel or scaffold, which can support the cells and mold them into the desired shape to obtain the desired function. Current advanced imaging technology, such as CT, enabled the creation of accurate CAD models for 3D printing to ensure a perfect fit into the desired tissue (58). Building various types of thick tissues with different shapes has been reported during the last few years with the ultimate target to print the whole organ or body parts for organ transplantation. Stem cells can be harvested from a transplant recipient, and printing them into a replacement organ could help bypass complications associated with organ transplants, such as long waits for a donor or immune rejection of the transplanted organ. Several breakthroughs in 3D tissue bioprinting were demonstrated recently to create organ-level structures including bone (59), cornea (60), cartilage (61), heart (62), and skin (63). Zhou et al. (64), constructed a patient-specific ear-shaped cartilage using expanded microtia chondrocytes and a biodegradable scaffold. The 3D-printed cartilage was used for the reconstruction of five microtia patients with satisfactory aesthetical outcome. Thick, vascularized cardiac patches and a cellularized human heart with a natural architecture were recently demonstrated (62). An omental tissue biopsy was taken from patients, and the cells were reprogrammed to become pluripotent stem cells and then differentiated to cardiomyocytes and endothelial cells. The bioink was formed by separately combining the two cell types with hydrogels for the cardiac tissue and blood vessels. Functional vascularized patches according to the patient's anatomy were demonstrated (Figure 4A). Among the various human tissues, skin was the focus of intensive research work, aiming to create a replacement of damaged (e.g., burned) skin and for wound healing and skin ulcer treatment purposes. Baltazar et al. (63) described implantable multilayered vascularized 3D-printed skin graft. The skin was constructed by employing one bioink that contains human foreskin dermal fibroblasts, human endothelial cells, and human placental pericytes suspended in rat-tail type I collagen to form the dermis followed by printing with a second bioink containing human foreskin keratinocytes to form an epidermis. In this structure, keratinocytes formed a multilayered skin barrier, while the endothelial cells and pericytes self-assembled into interconnected microvascular networks, which appeared to improve the keratinocyte maturation.

Table 2 lists some recent achieved 3D-bioprinted organs or functional tissues. Fabrication of fully developed vascularized organs would allow building functional/living human organ constructs suitable for surgical implantation. However, achieving this target is still facing many challenges, particularly post-processing remodeling associated with tissue fusion, retraction, and compaction of the printed soft-tissue construct (1). Therefore, blueprint tissue/organs cannot be directly derived from clinical scanning images. To get the desirable organ size and shape, CAD must include experimentally estimated coefficients of specific tissue compaction, retraction, and remodeling (1).

**Table 2:** Main bioprinting studies for regenerative medicine.

	**Printing methods**	**Description**	**Specific achievement**	**References**
Heart & cardiac patches	Microextrusion 3D bioprinting using 3D printer (regenHU, Villaz-Saint-Pierre, Switzerland)	Cells from an omental tissue biopsy are reprogrammed to become pluripotent stem cells differentiated to cardiomyocytes and endothelial cells, while the extracellular matrix is processed into a hydrogel. The two cell types were embedded in the hydrogels to form bioinks for the parenchymal cardiac tissue and blood vessel printing. A proof of concept of a cellularized human heart with a natural architecture was also demonstrated (Figure 4A)	Bioinks originated from the same patient, which would minimize the immune response after transplantation Whole-organ (heart) bioprinting was demonstrated	(62)
Blood vessels (vascular bypass grafts)	Microextrusion 3D bioprinting	Poly(ε-caprolactone) (PCL), low molecular weight chitosan (CS), and hydrogels (H) were integrated for building the grafts. PCL has been used for fabricating the scaffolds due to its excellent thermal stability and compatibility. Alginate and hyaluronic acid were used as a hydrogel matrix, while collagen type I was added to the matrix to increase the bioactivity properties of the hydrogel matrix	Endothelial cell line (HUVEC) was used	(68)
	In-house built microextrusion 3D printing device	An alginate–gelatin solution was used as a bioink material to construct vessel-like structures by employing new rotary forming device (Figure 4B)	A theoretical model was established to analyze the vessel thickness under different conditions. The vessel thickness cannot be adequately predicted by the theoretical model but by controlling the printing parameters (speeds)	(65)
Heart valve	Microextrusion 3D bioprinting using Fab@Home™ open-source, open-architecture RP platform (www.fab@home.org)	Hybrid hydrogels [based on methacrylated hyaluronic acid (Me-HA) and methacrylated gelatin (Me-Gel)] were utilized to bioprint heart valve conduits containing encapsulated human aortic valvular interstitial cells (HAVIC). HAVIC encapsulated within bioprinted heart valves maintained high viability and remodeled the initial matrix by depositing collagen and glyosaminoglycans	Cells in the hydrogel formulations maintained a high post-printing viability and fibroblastic phenotype	(69)
Bone	Indirect 3D printing of powder on a Z-printer 310 (Z Corporation, Burlington, MA, USA)	Biphasic calcium phosphate (BCP) consists of a mixture of hydroxyapatite (HA), and beta-tricalcium phosphate (β-TCP) matrices were bioprinted as a scaffold to induce ectopic bone formation by osteoblast seeding and/or addition of BMP-2	The bioprinted bone constructs were implanted subcutaneously in rats	(70)
	An integrated tissue-organ printer (ITOP)	Cell-laden hydrogel was deposited together with synthetic biodegradable polymers that impart mechanical strength to fabricate mechanically robust tissue constructs (bone, cartilage, & skeletal muscle). This was accomplished by designing multidispensing modules for delivering various cell types and polymers in a single construct. Incorporation of microchannels into the tissue constructs facilitates the diffusion of nutrients to printed cells	Mandible and calvarial bone, cartilage, and skeletal muscle were fabricated with recapitulated native structure	(42)
	Extrusion-based direct writing bioprinting	Two different GelMA-based hydrogels were synthesized (one supported vasculogenesis and the other supported osteogenesis). GelMA hydrogels containing different concentrations of VEGF were bioprinted into well-defined 3D architectures to create a gradient of vasculogenic factors. The bioprinting and incorporation of a rapidly degradable GelMA hydrogel resulted in the formation of a perfusable lumen with an endothelial lining at the center of the construct	Perfusable blood vessel inside a bioprinted bone-like tissue construct	(71)
	Microextrusion 3D Bioprinting	A porous hydroxyapatite scaffold was printed to mimic native bone through a multipass extraction process with the addition of osteoblast-like cells. The scaffold used is appropriate for graft without inflammatory reactions and bone formation after 8 weeks of implantation (Figure 4C)	Full osteointegration of the scaffold with the native tissue was observed after 4 and 8 weeks of implantation in rabbit model	(66)
	Direct ink writing using 600 μm custom-made nozzle	A glass-ceramic scaffold, with a dimension of 6 × 6 × 6 mm, was bioprinted mimicking cortical bone with scaffold of hexagonal pore shapes (450, 550, 900, and 1,200 μm) (Figure 4D)	The obtained strength is 150 times more than reported values for polymeric and composite scaffolds and five times more than reported values for ceramic and glass scaffolds	(67)
	Digital laser processing (DLP)-based 3D printing	Haversian bone-mimicking scaffold with integrated hierarchical haversian bone structure. The scaffold has the potential to induce osteogenic, angiogenic, and neurogenic differentiation *in vitro* and accelerated the in-growth of blood vessels and new bone formation *in vivo*	Effective delivery of osteogenic, angiogenic, and neurogenic cells, which exhibited favorable osteogenesis and angiogenesis	(72)
Cartilage	Simultaneous photopolymerization using a modified HP Deskjet 500 printer	Poly(ethylene glycol) dimethacrylate (PEGDMA) with human chondrocytes were printed to repair defects in osteochondral plugs (3D biopaper) in layer-by-layer assembly. Printed human chondrocytes maintained the initially deposited positions due to the simultaneous photopolymerization of surrounded biomaterial scaffold	Enhanced proteoglycan deposition was observed at the interface between printed biomaterial and native cartilage	(73)
	A hybrid inkjet printing/electrospinning system	Electrospinning of polycaprolactone fibers was alternated with inkjet printing of rabbit elastic chondrocytes suspended in a fibrin-collagen hydrogel in order to fabricate a five-layer tissue construct of 1 mm thickness cartilage	The chondrocytes maintained 80% viability more than 1 week after printing	(74)
Skin	Eight electromechanical dispensers mounted onto a 3-axis, high-precision robot stage which enables printing of multiple cell types and scaffold materials simultaneously	Keratinocytes and fibroblasts were used as constituent cells to represent the epidermis and dermis, and collagen was used to represent the dermal matrix of the skin. The 3D-printed constructs were cultured in submerged media conditions followed by exposure of the epidermal layer to the air–liquid interface to promote maturation and stratification	The morphology of the 3D-printed skin tissue closely mimics the *in vivo* human skin tissue	(75)
	4D bioprinting system (Organ Regenerator 4D)	Extracellular matrix (ECM) which derived from nano-fat consisting of supportive proteins, growth factors, and cytokines has been printed with bioinks to apply onto the chronic wound site	High wound healing rate with complete closure of wound of 2~5 weeks after membrane application	(76)
	Laser-assisted Bioprinting	Fibroblasts and keratinocytes embedded in collagen were printed in 3D as multicellular grafts analogous to native archetype and the formation of tissue	Successful formation of adhering and gap junctions	(77)
	Pneumatic-based microextrusion 3D bioprinting	An implantable multilayered vascularized skin graft is formed using 3D bioprinting using a bioink containing human foreskin dermal fibroblasts, human endothelial cells derived from cord blood human endothelial colony-forming cells, and human placental pericytes suspended in rat-tail type I collagen to form a dermis followed by printing with a second bioink containing human foreskin keratinocytes to form an epidermis	The human EC-lined structures inosculate with mouse microvessels arising from the wound bed and become perfused within 4 weeks after implantation	(63)
	Extrusion-based 3D bioprinting	Full thickness of the human skin model showing undulated morphology of epidermal rete ridges, architectural, mechanical, and biochemical functionalities	The epidermis–dermis junction was recapitulated in the 3D bioprinted skin tissue	(78)
Ear	Digital near infrared photopolymerization (DNP)-based 3D printing technology	Digital near infrared (NIR) photopolymerization (DNP) was used to spatially induce the polymerization of monomer solutions such that the subcutaneously injected bioink can be noninvasively printed into customized tissue constructs *in situ*	Ear-like tissue constructs with chondrification and a muscle tissue repairable cell-laden conformal scaffold	(79)
	The ear scaffold used a PCL mesh as an inner core, which was wrapped with PGA unwoven fibers and coated with PLA. Expanded microtia cartilages were dropped onto the PGA/PLA layer of the ear-shaped scaffold	Patient-specific ear-shaped cartilage is fabricated *in vitro* using expanded microtia chondrocytes, compound, and biodegradable scaffold. Different surgical procedures were employed to find the optimal approach for handling tissue-engineered grafts	Mature cartilage formation during 2.5 years for 5 reconstructed patients auricles	(64)
Liver	Custom-made inkjet 3D bioprinter	3D liver tissue is constructed using hepatocyte attachment and formation of the cell monolayer by interacting with the galactose chain of galactosylated alginate gel (GA-gel) with asialoglycoprotein receptor (ASGPR) of hepatocytes	Controlling cell polarity with galactosylated hydrogels	(80)
	Microextrusion 3D bioprinting	Primary hepatocytes with MSCs are used to support hepatocyte function and viability time in 3D structures	The 3D hepatic architecture showed a higher cell viability compared to the 2D system	(81)
Diaphragm	Regenova® bio-3D printer with cells only (Kenzan method)	Scaffold-free tissue patches composed of human cells are 3D printed with high elasticity and strength. The resulting tissue is cut into a patch for implantation. The patches were transplanted into rats with surgically created diaphragmatic defects	Complete integration of the graft with the native tissue Regeneration of muscle, neovascularization, and neuronal networks within the reconstructed diaphragms Rats survived for 710 days after implantation	(82)

### 3D Bioprinting of Organ Models for Drug Discovery

The current attempt in the translational medical research community is to focus more on complex human factors and conditions rather than relying on animal models. While the simplicity of the traditional *in vitro* models makes them robust and suitable for high-throughput research, unfortunately, they provide only little biological relevance to the complex biological tissues of the human body, which makes the technology gap between the lab models and industry/clinic adoptable models dramatically wide. Bioprinting paves the way for creating biomimetic structures and environment that support *in vivo*-like cell–cell and cell–matrix interactions with high-resolution vascularized tissue. Bioprinted tissue would represent powerful tools to provide physiologically relevant *in vitro* human organ models for drug toxicity assays and disease modeling that faithfully reproduce the complex human's key physiological aspects. Typically, organotypic bioprinting requires a large number of cells of different types to achieve a physiologically relevant heterotypic tissue, which renders it an expensive approach for large-scale and high-throughput assays. In addition, without a high-resolution vascularization that ensures long-term viability, a hypoxic environment may develop in the fabricated tissue due to the limited diffusion of cell nutrients into the core of the tissue. The integration of bioprinting and microfluidic technology provides an excellent opportunity to create miniaturized *in vitro* tissue models “organs-on-a-chip” that overcome these shortcomings. For example, various organotypic tissues can be simultaneously printed in a compartmentalized microfluidic chip and then connected through a vascular network (perfusion channels) to finally create multi organs on a chip “human-on-a-chip.”

It becomes generally accepted that 3D tissue models are superior and physiologically more relevant compared to the 2D countermodels. Furthermore, these tissue models are not subject to the rigorous ethical issues, which makes them an attractive choice for many relevant industries. However, they are still not systematically validated for toxicity prediction. To enable these powerful models for high-throughput drug discovery, systemic validation and standardization are required to certain their potential value.

Over the past few years, several companies and start-ups have launched 3D tissue *in vitro* models for toxicity screening and disease modeling. For example, Organovo Inc. developed a bioprinting process that can be tailored to produce tissues in various formats, including microscale tissues in multi-well tissue culture plates. For instance, human primary hepatocytes, hepatic stellate cells, and endothelial cells were used to bioprint liver-like tissue constructs. The tissue model was utilized to monitor the tissue response to amethotrexate and thioacetamide exposure, such as a liver injury that leads to fibrosis (83). In another study, Kupffer cells were added to examine their impact on the injury/fibrogenic response following cytokine and drug stimuli (84).

**Figure 5:**
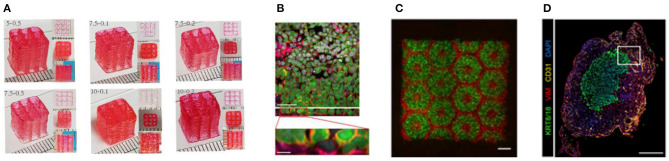
**(A)** High aspect ratio printed structure using layer-by-layer UV-assisted technology. Reproduced from Zhuang et al. (96) (open access, CC-BY). **(B)** Air–blood tissue barrier analog composed of an endothelial cell (HUV-EC cell line), basement membrane, and epithelial cell layer (A549 cell line). Reproduced from Horvarth et al. (40). Open access (BB-CY). **(C)** A 3D hydrogel-based triculture model that embeds hiPSC-HPCs with human umbilical vein endothelial cells and adipose-derived stem cells are created a microscale hexagonal architecture. Reproduced from Ma et al. (88) with permission from PNAS. **(D)** Multiple cell types were incorporated into scaffold-free tumor tissues with defined architecture. The technique enables modeling patient-specific tumors by using primary patient tissue. Reproduced from Langer et al. (93). Open access (BB-CY).

The rapid advances in bioprinting technology and the wide spread of the 3D bioprinter modalities have sparked unprecedented interest in using this technology to produce *in vitro* models in pharmaceutical research. Table 3 lists some selected examples of using the 3D bioprinting technology in fabricating *in vitro* models of tissues/organs for *in vitro* drug screening. Bowser and Moore (95), constructed a neural microphysiological system by employing spheroid and magnetic-based 3d bioprinting technology. Spinal cord spheroids, fabricated using magnetic nanoparticles, are positioned in a three-dimensional hydrogel construct using magnetically assisted bioprinting method. The constructs demonstrated localized cell–cell interactions and long-distance projections that mimic the *in vivo* structure. Zhuang et al. (96), combined the extrusion-based bioprinting technique with an in-built ultraviolet (UV) curing system to enable layer-by-layer UV curing of bioprinted photo-curable GelMA-based hydrogels. Using this technique, high aspect ratio and stable cell-laden constructs were achieved without the need of using reinforcement materials such as poly(-caprolactone) (PCL) polymer within the 3D-bioprinted constructs (Figure 5A). A recent study by Grigoryan et al., proved that food dyes could serve as potent photoabsorbers for the production of cytocompatible hydrogels with functional vascular topologies (85). Using this approach, they demonstrated functional vascular topologies for various studies (Figure 5B). Another study by Heinrich et al. demonstrated the construction of mini brains consisting of glioblastoma cells and macrophages as tool for testing therapeutics that target the interaction between these two cell types (92). A hybrid 3D cell-printing system was developed which utilized both the extrusion-based and inkjet-based dispensing modules to print a 3D human skin model within a transwell system (97). A collagen-based construct with polycaprolactone was printed using extrusion-based printing, and the inkjet-based dispensing module was used to uniformly distribute the keratinocytes onto the engineered dermis. 3D intestinal tissue was also bioprinted using human primary intestinal epithelial cells and myofibroblasts to model the architecture and function of the native intestinal tissue. The tissue model showed key anatomical and physiological characteristics such as a polarized epithelium with tight junctions and expression of CYP450 enzymes (98).

**Table 3:** Main bioprinting studies for *in vitro* models for drug discovery.

**Printed tissue or organ**	**Printing methods**	**Description (cell/bioink)**	**Stimuli/effect**	**Ref**.
Air-blood barrier	Laser-assisted 3d bioprinting with a printing resolution of 5 μ m)	Air–blood tissue barrier analogy composed of an endothelial cell (HUV-EC cell line), basement membrane, and epithelial cell layer (A549 cell line) (Figure 5B)	Cellular morphology, cell–cell contacts, and viability	(40)
Multivascular networks	Stereolithography	Intravascular and multivascular networks are fabricated with photopolymerizable hydrogels by using food dye additives as biocompatible but potent photoabsorbers for projection stereolithography	Oxygenation and flow of human red blood cells during tidal ventilation and distension of a proximate airway	(85)
Muscle & tendon tissues	Laser-assisted bioprinting (RegenHU, Switzerland)	Musculoskeletal-tendon-like tissue structures were 3D printed with alternating layers of photo-polymerized gelatin-methacryloyl-based bioink and cell suspension (primary human skeletal-muscle-derived cells and primary rat-tail tenocytes) in 24-well plates	Electrical stimulation and calcium signaling	(86)
Liver tissues	Microextrusion-based bioprinting (NovoGen Bioprinter)	A liver tissue-like structure that comprises primary human hepatocytes, hepatic stellates, and HUVEC cells in a defined architecture is 3D printed	Drug (Trovafloxacin)-induced liver injury	(87)
	Custom-built bioprinting system based on digital micro-mirror device with motion controller (Newport) that controls a movable stage	A 3D hydrogel-based triculture model that embeds hiPSC-HPCs with human umbilical vein endothelial cells and adipose-derived stem cells created a microscale hexagonal architecture (Figure 5C)	Liver-specific gene expression levels, increased metabolic product secretion	(88)
Skin	Freeform fabrication technique, based on direct cell dispensing using four pneumatically driven microvalves as dispensers and a three-axis robotic stage	Multilayered tissue composites, which consist of human skin fibroblasts and keratinocytes, are printed using a robotic platform that prints collagen hydrogel precursor, fibroblasts, and keratinocytes. The cell-containing collagen was cross-linked by coating the layer with nebulized aqueous sodium bicarbonate	Multilayered cell–hydrogel composites printing on a non-planar surface skin wound repair modeling	(89)
	Extrusion-based bioprinting	Skin is printed with a thickness of 5 mm using a bioink that was formulated as a mixture of bovine gelatin, very low viscosity alginate, fibrinogen, and human dermal fibroblasts	Bioink properties	(90)
	Extrusion-based bioprinting	Skin tissue equivalents in a multi-well plate format printed using neonatal human dermal fibroblasts and neonatal normal human epithelial keratinocytes	Barrier function (permeability tracing with Lucifer yellow and biotin tracer)	(91)
Mini brain	Extrusion-based bioprinting	Mini brains consisting of glioblastoma cells and macrophages are bioprinted as a tool to study the interactions between the two cell types and to test therapeutics that target this interaction. A two-step bioprinting process was used in which we first print the larger brain model encapsulating a mouse macrophages cell line (RAW264.7) with an empty cavity was printed, which in the second step is filled with mouse glioblastoma cells (GL261) embedded into bioink, followed by photo-cross-linking of the construct	Macrophages induce glioblastoma cell progression and invasiveness in the mini brains	(92)
Tumor breast & pancreatic	Microextrusion-based bioprinting (NovoGen Bioprinter)	Multiple cell types were incorporated into scaffold-free tumor tissues with defined architecture. The technique enables modeling patient-specific tumors by using primary patient tissue (Figure 5D)	Cellular proliferation, ECM deposition, and cellular migration are altered in response to extrinsic signals or therapies	(93)
	Laser direct write (LDW) bioprinting	Cell-encapsulating microbeads were generated and further processed into core-shelled structures, allowing for the growth and formation of self-contained, self-aggregating cells (e.g., breast cancer cells, embryonic stem cells)	The impact of aggregate size on the uptake of a commonly employed ligand for receptor-mediated drug delivery, transferrin	(94)

Although various tissues/organ models have been envisioned and manufactured, the level of complexity needed to make physiologically relevant tissue and organ replacements/models is still not achieved or clearly defined. *In vivo*, multiple cell types contribute to tissue development and homeostasis in well-connected tissue and organs within the biological systems. The inherent complexity of interconnected human tissues and animal models makes it difficult to mimic their structure and physiology to enable tracking the physiological events. Until now, it is not clear what level of biomimicry of human physiology is needed and whether we need to use all the cellular subpopulations to achieve differentiation into the needed phenotypes (9). Recent advances in 3D bioprinting technology show great potential to answer these critical questions. For example, complex heterogeneous cellular structures can be fabricated with multimaterial depositing systems (99, 100), hence enabling the incorporation of vascular and neural networks within the structure of the *in vitro* models, thus capturing the complexity of multiple tissue and organ systems. However, to achieve this ambitious aim, more profound and comprehensive studies are needed. In addition, integration of the bioprinting techniques with other technologies such as imaging, bioreactor technology, organ-on-a-chip (OOC), artificial intelligence (AI), and semiconductors would expand tissue engineering capabilities and accelerate the technology maturation toward organ/tissue production for various applications.

### *In situ* Bioprinting

**Figure 6:**
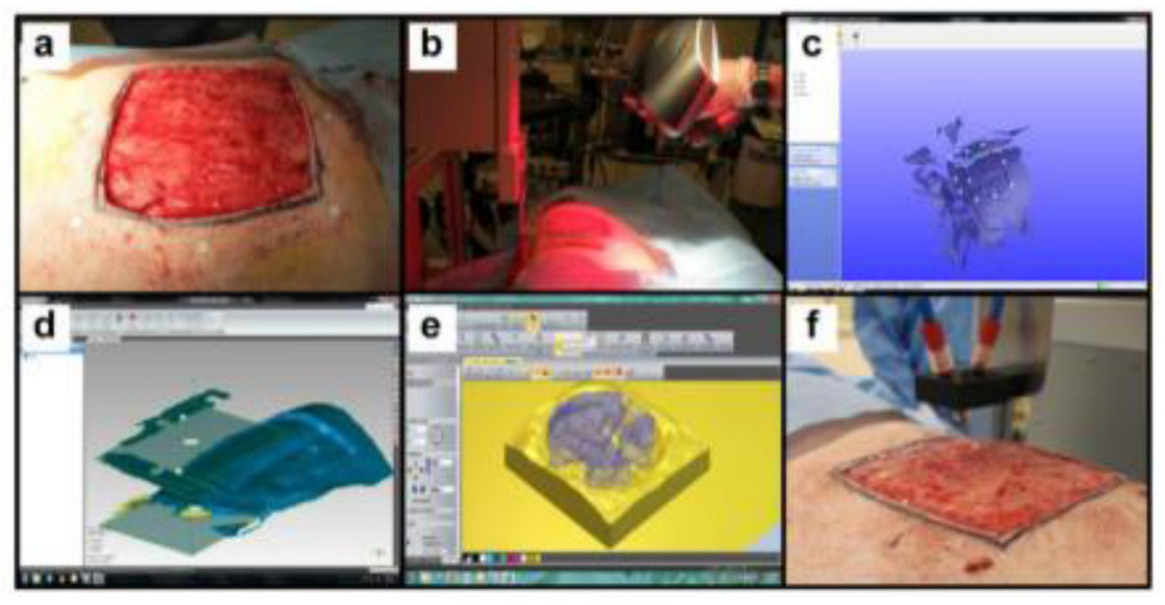
*In situ* skin bioprinting process. The skin area of interest is scanned with a handheld ZScanner™ Z700 scanner then the image is converted to an STL file; the scanned data is used to generate the fill volume and the path points for nozzle head movement; an output code is then provided to the custom bioprinter control interface for generation of a nozzle path needed to print the fill volume [reproduced from Albana et al. (101), open access (CC-BY)].

One of the promising applications of 3D bioprinting is to pattern *de novo* tissue directly onto the desired location in the body, such as chronic wounds in the skin or bone defect. With the aid of medical imaging, the topology of printed tissue can be designed to fit into the wound/defects such that heterotypic cellular structures, hydrogels, and soluble factors can be precisely deposited inside the defects. This approach, termed as *in situ* bioprinting or intraoperative bioprinting (IOB), would minimize the gap between implant–host interfaces and provide well-defined structures within zones of irregular topographies during the healing process, which can effectively recruit desired cells from surrounding tissues where the patient's body act as a natural bioreactor (5). Compared to the other applications listed above, only a few attempts have been reported. In a recent proof-of-concept study, Albana et al. (101) demonstrated precise delivery of autologous/allogeneic dermal fibroblasts and epidermal keratinocytes directly into an injured area in animals, replicating the layered skin structure (Figure 6). Excisional wounds bioprinted with layered autologous dermal fibroblasts and epidermal keratinocytes in a hydrogel carrier showed rapid wound closure, reduced contraction, and accelerated re-epithelialization. These results showed the feasibility of *in situ* bioprinting of skin and its potential applications for the regeneration of various body parts. A successful *in situ* bioprinting technique could rapidly accelerate healing using cell therapy, where cells can be isolated from a small biopsy (103). Zhao and Xu (102) developed a micro-bioprinting system installed to an endoscope to enable bioprinting inside the human body and utilized printed circuit micro-electro-mechanical-system techniques that allow a high-accuracy tissue printing. Two-layer tissue scaffolds were printed in a stomach model using gelatin–alginate hydrogels with human gastric epithelial cells and human gastric smooth muscle cells as bioinks to mimic the anatomical structure of the stomach. Kérourédan et al. (104) employed laser-assisted bioprinting (LAB) to pattern endothelial cells into a mouse calvaria bone defect, which is filled with collagen-containing mesenchymal stem cells and vascular endothelial growth factor. This technique enabled organized microvascular networks into bone defects with promising vascularization rate for *in situ* prevascularization that promote bone regeneration.

*In situ* bioprinting is a contact-based technique that requires special consideration compared to *in vitro* printing including bioink properties, bioprinter setup, and sterilization (105). For example, in extrusion-based bioprinting, the printing tip might interfere with the defect surrounding and caused side-effect damage. Generally, the bioinks used for *in situ* bioprinting need to be biocompatible with rapid cross-linkability to enable shorter surgery time and to retain the integrity of bioprinted constructs. Several biomaterials show high potential for such purposes, such as collage, fibrinogen, gelatin methacrylamide (GelMA), hyaluronic acid methacrylate (HAMA), and poly (ethylene glycol) (106). Vascularization is a major challenging task particularly for *in situ* bioprinting since it takes more than 10 days for angiogenesis to take place in living tissue (107). Temporal oxygen supply can be used prior to angiogenesis by using oxygen-generating biomaterials or oxygen-filled microparticles which can be bioprinted within the bioink (108, 109). Another strategy involves the creation of sacrificial porous structure within the bioprinted tissue by using meshed filaments (110).

### Bioprinting Meets Microfluidics and Organ-On-A-Chip

Recent bioprinting studies leveraged the well-established microfluidic technology to design bioprinting systems that enable precise dispensing of low-viscosity bioink in a well-defined template with highly controlled conditions (111–113). Microfluidic dispensing technology has been adopted in some commercial bioprinters. For example, Aspect Biosystems developed RX1TM bioprinter, which enables precise motion and pressure control that allows microscale resolution at high speed [https://aspectbiosystems.com/technology#bioprinter]. Abelseth et al. (114) reported using the RX1TM bioprinter to create 3D neural tissues derived from hiPSC-derived neural aggregates. The ability to create scaffolds with complex 3D shapes would enable precise control of the microstructures and microarchitecture of tissue constructs and hence the fabrication of various tissues and organ as *in vitro* models for drug discovery. To create miniaturized *in vitro* models of human organs, also known as organ-on-a-chip and organoids, one option is to follow a bottom-up approach by spatially immobilizing various types of living cells to generate heterogeneous functional structures within a prefabricated chip and scaffold. This approach also would enable the creation of more complex heterogeneous tissue structures, i.e., multiorgans-on-a-chip. 3D printing technology fits well in this specific domain and could be used to bridge critical gaps in tissue engineering. Various miniaturized organ models have been recently realized (i.e., printed) including liver (115), heart (116), vasculature (85), and kidney (117). Human cell-based organoids have become promising tools for drug screening and personalized medicine and disease modeling. Utilizing these organoids as building blocks in 3D bioprinting would enable scaling up the deposition of these tissue constructs. Maloney et al. (118) described an immersion printing technique to bioprint tissue organoids in 96-well plates. To maintain a spherical shape, hyaluronic acid and collagen-based hydrogel is bioprinted into a viscous gelatin bath, which prevent the bioink from interacting with the well walls. Reid et al. (119) used 3D bioprinting to create tumoroid arrays for studying the tumorigenesis and microenvironmental redirection of breast cancer cells. It was shown that adopting the bioprintering methodology significantly increases tumoroid formation in 3D collagen gels and allows precise generation of tumoroid arrays as well as co-printing cancer cells with epithelial cells to generate chimeric organoids. The integration of 3D bioprinting, 3D cell culture, microfluidics, and organ-on-a-chip has great potential to enable the integration of multiple organoids within a single system with small footprints and improved biosensing capability.

### 3D Bioprinting for Animal-Free Meat

Animal proteins represent 40% of total global protein consumption (120). While the demand for animal protein is expected to be doubled by 2050 associated with the increase of the global population (121), current livestock production is facing several problems such as pollution, shrinking of animal habitat, increased soil erosion, and greenhouse gas emissions (122). Many researchers are now proposing to shift toward more sustainable meat resources such as *in vitro* meat (IVM) production, which becomes the subject of extensive media coverage.

**Figure 7:**
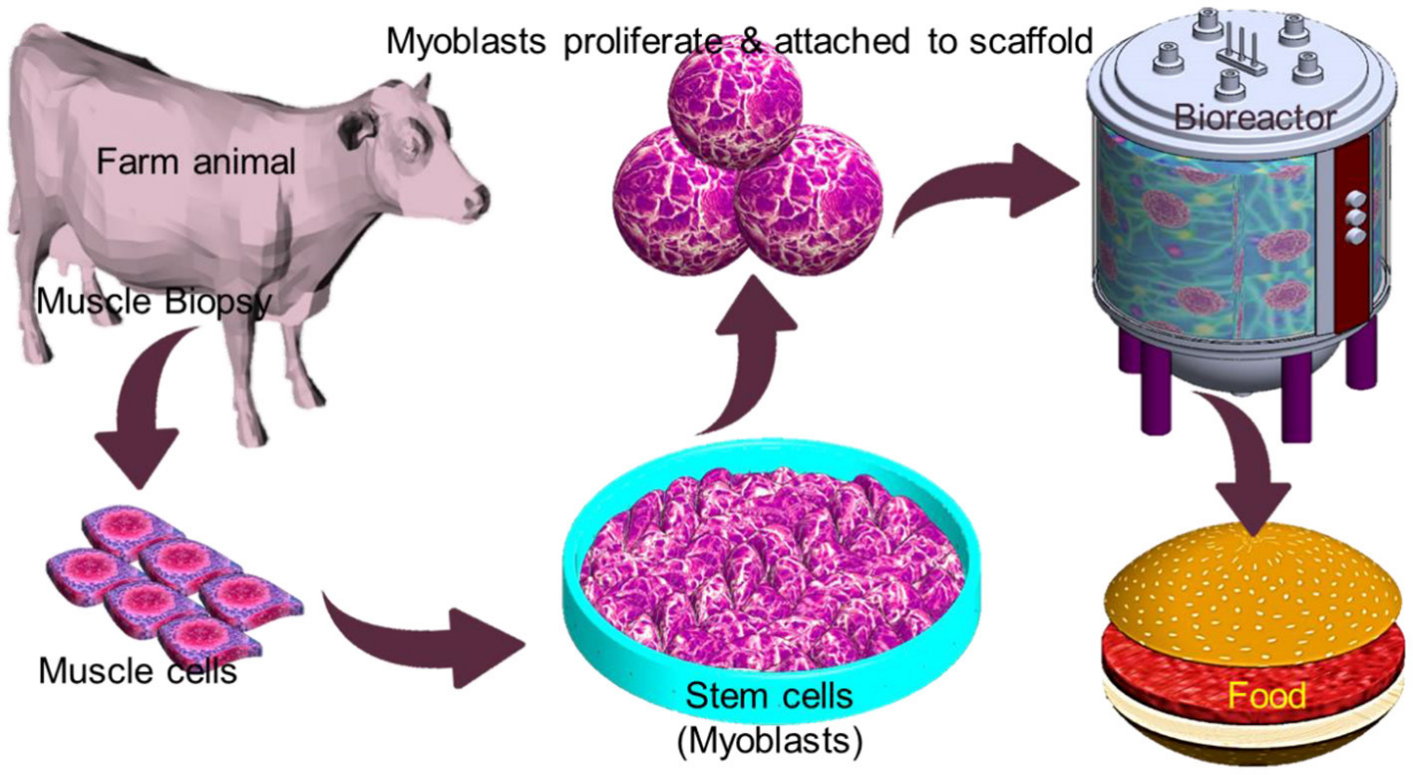
Overview schematic of the *in vitro* meat production process.

IVM production offers a safe way to meet the increasing demand for protein without involving animal sacrifices and reducing the impact of the aforementioned issues. However, associated with high production cost, public neophobia may limit its commercial viability in the near future (123). Conventional edible meat mainly consists of skeletal muscles along with adipocytes, fibroblasts, and endothelial, which give it a nutritional value. The technique to generate muscle tissues *in vitro* relies on various cell types for initiating the production of meat, with the most promising being myosatellite cells, which are the primary adult stem cells for muscle (124). Myosatellites are separated from a biopsy that is taken from a suitable animal and cultured in a proper culture condition that involves a continuous supply of nutrients and growth factors to induce multinuclear myotube growth. Maturation of myotube and further growth by continued differentiation and merging of new myoblasts results in the formation of muscle fibers (Figure 7). A key requirement of tissue engineering involves a scaffold to support cell proliferation. Similarly, myoblast proliferation also requires a flexible scaffold with a large surface area that can be easily dissociated from the final meat product and enable contraction and maximize medium diffusion (125). Alternatively, the scaffold material needs to be natural-based and edible. A major challenge in IVM is to define food-grade culture media that is affordable in large quantities. Animal-based sera have been used as standard supplements for cell culture media for decades. However, adopting this methodology raises ethical and regulatory concerns. Alternatively, plant-based growth media substitution may eliminate the controversial animal-based growth media^2^. In order to be accepted by the end customer, the nutritional value of the *in vitro* produced meat must be equivalent to or higher than that of conventional meat. It is noteworthy that *in vitro* meat can be supplemented with even desired nutrients such as vitamins and minerals (126).

The biggest technical challenge for the IVM industry lies in scaling up the product toward commercialization. The current price of lab-grown meat is extremely high, which hinders its commercial value. However, with the advance of bioreactor technology, the last few years witnessed a decline in the prices, which is a good sign for commercialization. The primary obstacles holding back the IVM technology from scaling up are the high-cost culture medium and microcarriers and implementing the suitable large-scale bioreactor for mass production. Finally, public acceptance of IVM needs to be preceded by regulatory standards and guidelines that bring comfort among consumers and reduce skepticism among start-ups working in the field.

## Challenges and Limitations of the 3D Bioprinting Technology

The ultimate aim of 3d bioprinting is to develop a technology that will be able to realize 3D functional complex organs as a source for tissue grafts, full-organ transplants, and animal-alternative models for drug screening. This technology is still in the very early stage but rapidly moving forward with a plethora of research span from printing engineering to tissue engineering and cell sciences being done on bioprinting technology.

Despite the significant progress and many breakthroughs, bioprinting technology is still facing several serious challenges that delay scaling up bioprinting structures to viable and functional tissues. The greatest challenge is the ability to print an intra-organ vascular hierarchical network, from arteries and veins down to capillaries, without which tissues will not survive. *In vivo*, a vasculature network is required for tissues to grow beyond 100–200 μm (127) as this is the diffusion limit of oxygen (128). The fabrication of large tissue segments with a high volumetric oxygen-consumption rate, such as cardiac and liver tissues, would require adequate oxygen supply to prevent a shortage in nutrient and tissue necrosis. Fabricating blood capillaries is currently restricted due to the current limitation of 3D printing resolution, which is ~20 μm, while the blood capillary can be as small as 3 μm. Several promising solutions are being exploited to create vascularized human tissue, for instance, by incorporating angiogenic growth factors into bioinks to induce vasculature growth after printing (129, 130). The combination of 3D bioprinting and self-assembly of microvascularized units as building blocks was proposed by Benmeridja et al. (131). In this study, adipose-derived stem cells and human umbilical vein endothelial cells (HUVEC) were cocultured with favorable seeding technique and conditions to enable the formation of compact viable adipose tissue spheroids with capillary-like network. Another approach used a microfluidic device to induce vasculogenesis (132); however, the used hydrogels do not support cell–cell interactions and affect phenotypic stability (3). Owing to the complexity and small size of the vasculature network, developing functional vasculature in a timely manner to support the bioprinted tissue is still not achievable to date.

Biomaterials play a primary role in 3d bioprinting for supporting the structural and functional features of the printed tissue and maintaining the structural integrity and biocompatibility during tissue printing and maturation. However, current available printable materials are not capable of fully mimicking the native ECM compositions to support the cellular structure. Therefore, it is crucial to develop new printable biomaterials that can be printed together with live cells and possess adequate mechanical properties for cell handling.

Cell sourcing is another great challenge, as tissue printing requires a large number of cells. Stem cell source would be the most promising choice as bioprinting would influence stem cell differentiation at multiple stages of the process. Another current limitation of 3D bioprinting is the low throughput and high cost. All current techniques require manual cell seeding and bioink loading while high-throughput production of 3D models is in demand. 3D organoids offer in the future a good large-scale screening tool for drug discovery. However, organoids are still produced in small-scale tissue culture plates. In an attempt to demonstrate high-throughput bioprinting, Hwang et al. (133) reported rapid fabrication of complex 3D live hepatocellular carcinoma 3D tissue scaffolds in multiwell plates for subsequent culture and analysis. The bioprinted tissue samples were then used to test drug response against the chemotherapy drug doxorubicin. Also, the production costs is another major problem since many expensive reagents (e.g., growth factors) are required. To address these issues, trans-disciplinary research involving cell biologists, engineers, physiologists, and pharmaceutical industry partners is necessary to enable and push the boundaries of this technology.

## Future Perspectives

With advances in tissue engineering, the possibility of regenerating *de novo* tissue or organs *in vitro* has become a real matter for the first time in medicine history. Despite many challenges, the successful demonstration of printable tissue structures during the last decade is a good sign for a very fascinating and promising approach in various medical and industrial application domains, which is worth more investigation.

The ultimate goal of 3D bioprinting technology is to enable industry-scalable printing of functional living tissue/organ. Many significant works on bioprinting processes, materials, and related technologies were demonstrated, which show the high momentum toward achieving this goal. However, despite the significant progress in every individual-related technology, it is very important to integrate these technologies together, to establish the necessary standards and to enable process automation/robotization to eventually enable scalable industrial organ printing.

Tissue engineering is transdisciplinary, and in order to push the bioprinting-based tissue engineering beyond the laboratory, comprehensive, and systematic studies by engineers, scientists, and clinicians on bioink optimization, bioreactor engineering and cell culture environment are critically needed to enable high-throughput production that is associated with efficient screening assays. Because the living tissue/organ structure is very complex, to reproduce them *in vitro*, it is required to develop printing tools which are able to print hybrid materials (bioinks) with high resolution, speed, and maintained biocompatibility and reproducibility. This could be achieved by integrating the bioprinting technologies with other enabling techniques such as 3D cell culture, bioreactor technology, microfluidics, and organ-on-a-chip. With suitable bioinks, supported by advanced biofabrication technologies, this technology will allow bridging the currently huge existing gap between the lab and fab to ultimately meet the current clinical and industrial needs and push the boundaries for advanced drug discovery and regenerative medicine.

3D bioprinting could present a paradigm shift for the 21st century in many biomedical sectors. To accelerate the advances of this technology and turn this vision into reality, effective collaboration and information dissemination between the scientific and engineering community becomes a crucial necessity.

## Author Contributions

All authors listed have made a substantial, direct and intellectual contribution to the work, and approved it for publication.

## Conflict of Interest

The authors declare that the research was conducted in the absence of any commercial or financial relationships that could be construed as a potential conflict of interest.

## Footnotes

^1^Available online at: https://www.ddw-online.com/therapeutics/p316729-spheroids-rapidly-becoming-a-preferred-3d-cell-culture-format.html

^2^ https://www.minitube.com/products/camelids/semen-extenders/andromed-200-ml
